# Stem Cells From Human Exfoliated Deciduous Teeth Alleviate Liver Cirrhosis *via* Inhibition of Gasdermin D-Executed Hepatocyte Pyroptosis

**DOI:** 10.3389/fimmu.2022.860225

**Published:** 2022-05-12

**Authors:** Peng Chen, Yi-kun Zhou, Chun-shan Han, Liu-jing Chen, Yi-ming Wang, Zi-meng Zhuang, Shuai Lin, Yan-heng Zhou, Jiu-hui Jiang, Rui-li Yang

**Affiliations:** ^1^Department of Orthodontics, Peking University School and Hospital of Stomatology, Beijing, China; ^2^National Clinical Research Center for Oral Diseases and National Engineering Laboratory for Digital and Material Technology of Stomatology, Beijing, China; ^3^Beijing Key Laboratory of Digital Stomatology, Beijing, China

**Keywords:** liver cirrhosis, stem cells from human exfoliated deciduous teeth, NLRP3, GSDMD, pyroptosis

## Abstract

Liver cirrhosis represents a type of end-stage liver disease with few effective therapies, which was characterized by damaged functional liver tissue due to long-term inflammation. Gasdermin D (GSDMD)-executed programmed necrosis is reported to be involved in inflammation. However, the role of GSDMD in liver cirrhosis remains unclear. In this study, we used a CCl_4_-induced cirrhosis model and found stem cells from human exfoliated deciduous teeth (SHED) infusion showed profound therapeutic effects for liver cirrhosis. Mechanistically, NLRP3 inflammasome-activated GSDMD and its pyroptosis were upregulated in liver cirrhosis, while SHED infusion could suppress the expression of GSDMD and Caspase-1, resulting in reduced hepatocyte pyroptosis and inflammatory cytokine IL-1β release. Consistently, SHED could inhibit the elevated expression of NLRP3, GSDMD and Caspase-1 induced by CCl_4_ treatment *in vitro* co-culture system, which was mediated by decreasing reactive oxygen species (ROS) generation. Moreover, the pyroptosis inhibitor disulfiram showed similar therapeutic effects for liver cirrhosis as SHED. In conclusion, SHED alleviates CCl_4_-induced liver cirrhosis *via* inhibition of hepatocytes pyroptosis. Our findings could provide a potential treatment strategy and novel target for liver cirrhosis.

## Introduction

Liver cirrhosis is prevalent worldwide and is associated with high morbidity and mortality (1). Cirrhosis is the result of chronic liver inflammation, wherein the normal liver structure is replaced by fibrotic liver nodules and finally leads to liver failure (2). The current drugs and immunotherapy failed to cure patients with liver cirrhosis. Liver transplant is still the most effective cure for liver cirrhosis. However, few patients benefit from organ grafting due to donor organ shortage and the need for lifelong immunosuppressive therapy (3). It is urgent to explore potentially new therapeutic strategies.

Mesenchymal stem cells (MSCs) showed profound immunomodulatory effects except for the capacity for self-renewal and multi-lineage differentiation (4). Recent studies showed that MSCs demonstrated promising therapeutic for several immune diseases, including asthma (5), multiple sclerosis (6), and COVID-19 (7). MSCs are considered to be a feasible cell source, which can be isolated from multiple tissues, such as bone marrow (8), adipose tissue (9), periodontal ligament (10), and dental pulp tissue (11). Among them, stem cells from human exfoliated deciduous teeth (SHED) are relatively new and less studied than MSCs from other sources. SHED is easily obtained and relatively free from ethical concerns compared with MSCs derived from other tissues. SHED exhibited a stronger angiogenesis differentiation, proliferation potential (12), and anti-apoptotic ability over bone marrow MSCs (BMMSCs) (13). SHED was reported to attenuate liver fibrosis by differentiating into hepatocyte-like cells *in vivo* (14), and SHED-derived hepatocyte transplantation also eliminated liver fibrosis (15). Our previous study showed that SHED could prevent acute hepatitis *via* inhibition of hepatocyte apoptosis (16). However, the effect of SHED infusion on liver cirrhosis and the underlying mechanism remains elusive.

Pyroptosis is a lytic type of cell death characterized by the activation of inflammatory caspases. Pyroptosis is found to play important roles in a variety of inflammatory diseases, such as sepsis (17), tuberculosis (18), HIV (19), and some neural diseases (20). Gasdermin D (GSDMD) has been identified as the sole effector of pyroptosis. Recently, it is reported that NLR family CARD domain-containing protein 4 (NLRC4) inflammasome was found to mediate the pyroptosis of hepatocytes in non-alcoholic steatohepatitis (NASH) (21). However, whether pyroptosis is involved in liver cirrhosis and the specific effects is still unknown.

In the present study, we evaluated the therapeutic effect of SHED on CCl_4_-induced mouse liver cirrhosis and revealed that SHED administration attenuated liver cirrhosis by alleviating GSDMD-mediated pyroptosis and inflammation.

## Materials and Methods

### Animals

Female C57BL/6N mice aged 6–8 weeks (body weight 20–21 g) were purchased from Vital River Laboratory (Beijing Vital River Laboratory Animal Technology, Beijing, China). The mice were provided standard chow and water and housed at 22°C–24°C for 1 week before the experiment. The study was authorized by the Peking University Animal Ethics Committee (No. LA2019077).

### Liver Cirrhosis Induction

To evaluate the therapeutic effects of SHED, the mice were randomly divided into 3 groups (n = 4): control, CCl_4_, and CCl_4_+SHED. A 20% solution of CCl_4_ in corn oil was injected intraperitoneally into C57BL/6J mice (5 ml/kg) twice a week for 4 weeks (22, 23), and then SHED (1 × 10^6^) suspended in 100 μl of phosphate-buffered saline (PBS) was injected by tail vein injection twice a week (14). The livers were harvested 2 months after induction of liver cirrhosis and treatment.

To evaluate the therapeutic effects of disulfiram, the mice were randomly divided into 3 groups (n = 4): control, CCl_4_, and CCl_4_+disulfiram (a Food and Drug Administration (FDA)-approved pyroptosis inhibitor). A 20% solution of CCl_4_ in corn oil was injected intraperitoneally into C57BL/6J mice (5 ml/kg) twice a week for 4 weeks (22), and then disulfiram dissolved in corn oil was injected intraperitoneally (10 mg/kg) once a week. The livers were harvested 2 months after induction of liver cirrhosis and treatment.

### Biochemical Indicator Analysis of Mouse Serum Blood Samples

The serum was separated to detect the biochemical indexes. Serum alanine aminotransferase (ALT) and aspartate aminotransferase (AST) were measured by Elisa kit (Changchun Huili Biotech, Changchun, China) according to the manufacturer’s instructions. They are commonly clinically measured as markers for liver dysfunctions. For the ALT and AST assays, 10 μl of serum was used.

### Optical Imaging

Mice were intravenously injected with prepared DiR-labeled SHED in PBS) and scanned at 1 and 3 days post-injection using an Interactive Video Information System Lumina Series III *in vivo* Imaging System (Caliper Life Sciences, PerkinElmer, Waltham, MA, USA). *Ex vivo* imaging was carried out immediately afterward by imaging excised major organs (heart, lung, liver, spleen, and kidney).

### Frozen Sections

SHED was labeled with 5-(and-6)-carboxy fluorescein diacetate succinimidyl ester (CFSE; Molecular Probes Biotec Co., Eugene, OR, USA) and diluted to 1 × 10^7^/ml for transplantation. The livers were flash-frozen at an optimum cutting temperature at 1 and 3 days post-injection. Nuclear staining was performed with diamidine phenyl indoles (DAPI) mounting medium (Vector Lab, Burlingame, CA, USA). The positions of CFSE-labeled SHED cells could be traced using a confocal laser scanning microscope (Zeiss, Thornwood, NY, USA).

### H&E Staining

The livers were harvested and then fixed in 4% paraformaldehyde for 24 h at 4°C. Having been paraffin-embedded and sectioned into 5-μm pieces, deparaffinization and rehydration of sections were done. Then sections were stained with H&E staining kits (Solarbio, Beijing, China) in accordance with the manufacturer’s instructions. Images were captured by an inverted microscope.

### Masson Trichrome Staining

The liver tissue sections were stained with modified Masson trichrome staining kits (Solarbio, China) in accordance with the manufacturer’s instructions. The level of liver fibrosis was evaluated by calculating the percentage of the blue region (collagen) by ImageJ software. Three fields of view were randomly selected from each specimen (n = 3) for semiquantitative analysis of the collagen/matrix content.

### Sirius Red Staining

Sirius red staining was carried out according to the instructions of the Sirius red staining kits (Novon Scientific, Beijing, China). The sections were stained with Sirius red for 1 h. For collagen deposition determination, three randomly selected fields from each slice were visualized and quantified using ImageJ software.

### Immunohistochemistry

The enzymatic antigen retrieval method was used with trypsin and proteinase K at a ratio of 1,000:1. Endogenous peroxidase blocking was done using hydrogen peroxide, while 5% bovine serum albumin (BSA) was used to reduce non-specific protein reactions. Primary antibodies GSDMD (1:600) (Abcam, Cambridge, UK; ab219800), Caspase-1 (1:600) (ProteinTech, Chicago, IL, USA; 22915-1-AP), and NLRP3 (1:300) (Thermo Fisher, Waltham, MA, USA; PA5-79740) were dissolved in blocking buffer (Zhongshan Golden Bridge, Beijing, China). The diluted primary antibody was added to each slide and incubated overnight at 4°C. After being washed three times with PBS to remove the redundant primary antibodies, the slides were incubated with secondary goat anti-rabbit lgG (Zhongshan Golden Bridge, China) at 37°C for 60 min. The color intensification was done with a DAB chromogen kit (Zhongshan Golden Bridge, China) and followed by hematoxylin staining. Three fields of view were randomly selected from each specimen (n = 3) for semiquantitative analysis, and the positive-staining cells were counted by ImageJ software.

### Cell Culture

Mouse hepatic cell line NCTC1469 was purchased from Saibai Biotechnology. NCTC cells were cultured in high-glucose Dulbecco’s modified Eagle medium (DMEM) supplemented with 10% fetal bovine serum and 1% penicillin and streptomycin at 37°C with 5% CO_2_. For the CCl_4_ treatment group, the final concentration of CCl_4_ was 10 mmol/L. CCl_4_ was added to the medium and cultured for 8 h for further analysis. SHED was obtained from Oral Stem Cell Bank (Beijing, China), and they were isolated from different normal exfoliated human deciduous incisors. The pulp was separated from the crown and digested in a solution of 3 mg/ml collagenase type I (Sigma, St. Louis, MO, USA) and 4 mg/ml of dispase (Sigma, USA) for 1 h at 37°C. Then cell suspensions were cultured in α-МЕМ supplemented with 15% fetal bovine serum and 1% penicillin and streptomycin and incubated at 37°C in a humidified atmosphere containing 5% CO_2_. Protocols and procedures were approved by the Ethical Guidelines of Peking University (PKUSSIRB-2013 11103). SHED from passages 4–6 was used. Co‐culture experiments were carried out *via* a transwell co‐culture system (3-μm pore diameter). NCTC cells were seeded in the lower compartments of 6‐well transwell plates (2 × 10^5^) or 24‐well transwell plates (5 × 10^4^) (Costar Corning, Tehama County, CA, USA). The ratio of SHED and NCTC was 2:1, and SHED was seeded in the upper chamber. These plates were then incubated at 37°C for 8 h for further investigation.

### Intracellular Reactive Oxygen Species Analysis

Reactive oxygen species (ROS) detection kits were purchased from Solarbio Company (Beijing, China). The cultured NCTC were collected as single-cell suspension and then incubated with 2,7‐dichlorofluorescin diacetate (DCFH-DA) in the dark at 37°C for 30 min. The DCFH-DA fluorescent probe oxidized by ROS was analyzed using a flow cytometer (BD Accuri C6, BD Biosciences, San Jose, CA, USA).

### Propidium Iodide Staining

Propidium iodide staining was performed using a propidium iodide (PI) staining kit (Nanjing kaiji Bio-Tek Corporation, Nanjing, China). The cells were incubated with a PI solution for 30 min, followed by counterstaining with DAPI. Then, images were taken under a fluorescence microscope. The cell death rate was counted by the ratio of red/blue fluorescence intensity using ImageJ software. Three visual fields were selected for each group and photographed under a fluorescence microscope.

### Determination of Mitochondrial Membrane Potential

Mitochondrial membrane potential (MMP) was determined by using MMP assay kits with JC-1 (Solarbio, China). JC-1 working solution measuring 1 μg/ml was prepared and incubated with cells at 37°C for 20 min. The level of MMP depolarization was identified by the red/green fluorescence intensity ratio. Three visual fields were selected for each group and photographed under a fluorescence microscope.

### Immunofluorescence Staining

NCTC cells were seeded on chamber slides and fixed with 4% paraformaldehyde. The chamber slides were incubated with primary antibodies GSDMD (Abcam, ab219800), Caspase-1 (1:200) (ProteinTech, 22915-1-AP), and NLRP3 (1:200) (Thermo Fisher, PA5-79740) at 4°C overnight and then treated with sheep anti-rabbit conjugated with fluorescein isothiocyanate (FITC) (1:200) for 1 h at room temperature. Finally, the slides were mounted with a mounting medium containing DAPI. Three visual fields were selected for each group and photographed under a fluorescence microscope. Image analysis was performed using ImageJ.

### Quantitative Real-Time PCR Assay

RNA was extracted from NCTC cells with TRIzol reagent (Sigma, USA), and 2 µg of RNA was reverse transcribed into complementary first-strand cDNA using cDNA synthesis kits (Takara Bio, Inc., Otsu, Japan). Real-time PCR quantification was performed using the SYBR Green Mix (Applied Biosystems, Foster City, CA, USA). Target gene expressions were calculated by their ratios to β-actin. The sequences of primers were as follows: Caspase-1 forward: 5′-AATACAACCACTCGTACACGTC-3′, Caspase-1 reverse: 5′-AGCTCCAACCCTCGGAGAAA-3′; GSDMD forward: 5′-TTCAGGCCCTACTGCCTTCT-3′, GSDMD reverse: 5′-GTTGACACATGAATAACGGGGTT-3′; IL-1β forward: 5′-TTCAGGCAGGCAGTATCACTC-3′, IL-1β reverse: 5′-GAAGGTCCACGGGAAAGACAC-3′.

### Western Blotting Analysis

Total protein was lysed in radioimmunoprecipitation assay (RIPA) buffer (Highly Efficient Solitaire, Solarbio, China) containing 100 μM of phenylmethylsulfonyl fluoride (PMSF) (Solarbio, China) and protease inhibitor cocktail (Abcam, Waltham, MA, USA). Proteins measuring 25 μg were loaded and separated into 4%–12% NuPAGE gel (Beyotime, Shanghai, China), and they were transferred to 0.2 μm of nitrocellulose membranes (Millipore, Billerica, MA, USA). Then 0.1% Tween-20 and 5% BSA were provided to block the membranes for 1 h, followed by overnight incubation with primary antibodies (1:1,000). The membranes were washed and incubated for 1 h in horseradish peroxidase (HRP)-conjugated secondary antibody at 1:5,000 (Zhongshan Golden Bridge, China). Super Signal West Pico Chemiluminescent Substrate (Thermo) and BioMax film (Kodak, Rochester, New York, USA) were used to detect the immunoreactive proteins.

### Statistics Analysis

Statistical analysis was performed with GraphPad Prism 9.0 software. Comparisons between two groups were analyzed using independent unpaired two-tailed Student’s t-tests, and comparisons among more than two groups were analyzed using a one-way ANOVA with the Bonferroni correction if the data did not meet the normality distribution assumption. p-Values <0.05 were considered statistically significant.

## Results

### Stem Cells From Human Exfoliated Deciduous Teeth Administration Attenuated CCl_4_-Induced Mouse Liver Cirrhosis

**Figure 1:**
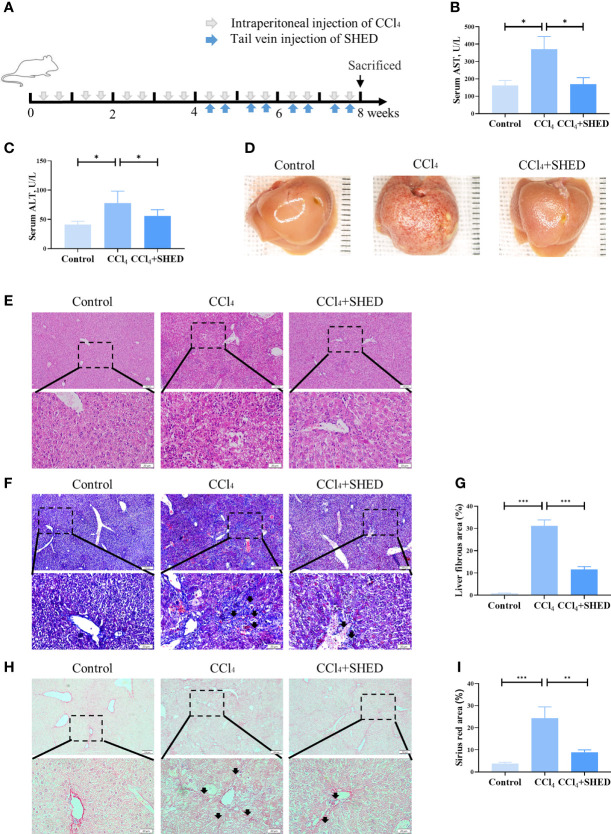
SHED alleviated CCl_4_-induced liver cirrhosis. **(A)** The schema of CCl_4_ injection to induce liver cirrhosis and SHED infusion in mice. **(B, C)** The levels of serum ALT and AST of control, CCl_4_, and SHED infusion groups. **(D)** The gross appearance of the liver tissues from control, CCl_4_, and SHED infusion groups. **(E)** H&E staining of liver tissues from control, CCl_4_, and CCl_4_ mice with SHED infusion groups. **(F, G)** The collagen deposition in livers from control, CCl_4_, and SHED infusion groups, as assessed by Masson trichrome staining. Arrow heads, fibrous deposition. **(H, I)** Sirius red staining of fibrous septum in livers from control, CCl_4_, and SHED infusion groups. Arrowheads, fibrous septum. Scale bar: 100 and 20 μm. *p < 0.05, **p < 0.01, and ***p < 0.001. Graph bars show the mean ± SD. All of the assays were performed in triplicate. ALT, alanine aminotransferase; AST, aspartate aminotransferase; CCl_4_, carbon tetrachloride; SHED, stem cells from human exfoliated deciduous teeth.

Mouse liver cirrhosis was induced by intraperitoneal injection of CCl_4_ for 4 weeks, and SHED (1 × 10^6^) cells were injected *via* tail vein to analyze their therapeutic effects (**Figure 1A**). Liver function was determined by ALT and AST levels, and the results showed that the level of ALT and AST was higher in the CCl_4_ group compared with the control one, and SHED administration significantly decreased the level of AST and ALT levels as compared with the CCl_4_ group (**Figures 1B, C**). The CCl_4_ group showed more fibrosis and effusion compared with the control group, while SHED infusion could significantly improve the morphology of the liver (**Figure 1D**). There were more swelling cells, inflammatory cells infiltration, and disorganized liver cells arrangement in the CCl_4_ group compared with the control group, and SHED treatment significantly ameliorated the swelling cells and the tissue inflammation, as assessed by H&E staining (**Figure 1E**). Moreover, Masson trichrome staining showed that collagen deposition was significantly increased in the CCl_4_ group, while SHED infusion could decrease the collagen deposition (**Figures 1F, G**). Sirius red staining showed that collagen fibers expanded outward from the portal area and formed fibrous septum in the CCl_4_ group, while SHED infusion reduced the formation of the fibrous septum (**Figures 1H, I**). These results indicated that SHED administration could reduce the CCl_4_-induced mouse liver injury.

### Stem Cells From Human Exfoliated Deciduous Teeth Infusion Inhibited Pyroptosis of Hepatocytes in CCl_4_-Induced Liver Cirrhosis

**Figure 2:**
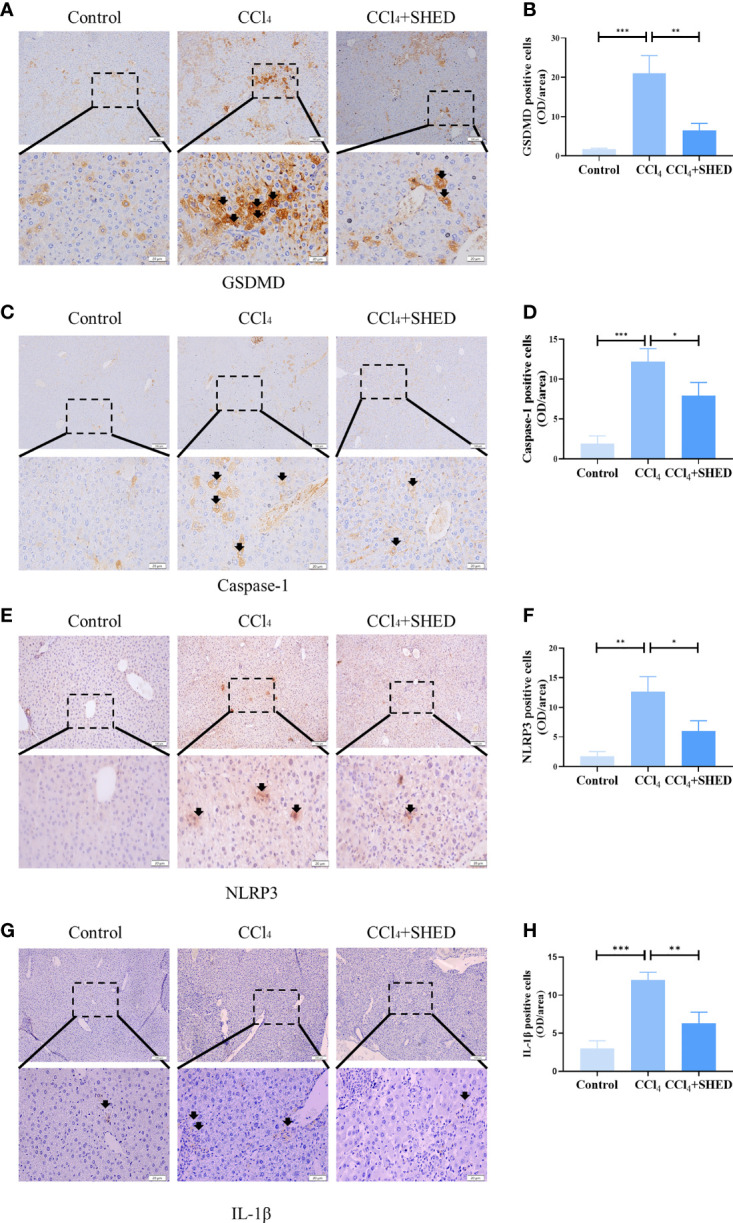
SHED attenuated pyroptosis of hepatocytes in CCl_4_-induced liver cirrhosis. **(A, B)** Immunohistochemical staining of GSDMD in control, CCl_4_, and SHED transplantation groups **(C–H)**. The expression of NLRP3, Caspase-1, and IL-1β in control, CCl_4_, and SHED transplantation groups, as assessed by immunohistochemical staining. Scale bar: 100 and 20 μm. *p < 0.05, **p < 0.01, and ***p < 0.001. Graph bars show the mean ± SD. All of the assays were performed in triplicate. NLRP3, NOD-like receptor family pyrin domain containing 3; GSDMD, gasdermin D; SHED, stem cells from human exfoliated deciduous teeth.

Pyroptosis plays important roles in inflammation (24). It is reported that hepatocyte pyroptosis may be involved in NASH (25, 26). The expression of GSDMD, a key pyroptosis executioner, was significantly increased in the CCl_4_-treated group compared with the control ones. With the treatment of SHED, the ratio of GSDMD positive cells was significantly decreased (**Figure 2A, B**). Then we analyzed the expression of Caspase-1 and NLRP3, the upstream molecules of GSDMD, and inflammatory cytokines IL-1β in liver tissue. Results showed that the expression of Caspase-1, NLRP3, and IL-1β was significantly upregulated in the CCl_4_ group. With the infusion of SHED, the expression of Caspase-1, NLRP3, and IL-1β was decreased (**Figures 2C–H**). NLRP3–Caspase-1–GSDMD is known as a classical pathway of pyroptosis (27). After NLRP3 activation, pro-IL-1β is cleaved into mature and active IL-1β mediated by Caspase-1, and active IL-1β is secreted extracellularly by the GSDMD pore to exert an inflammatory effect (28). These results indicated that SHED infusion attenuated liver cirrhosis through inhibited pyroptosis.

### Stem Cells From Human Exfoliated Deciduous Teeth Inhibited NLRP3 Inflammasome Activation and Pyroptosis in Hepatocytes

**Figure 3:**
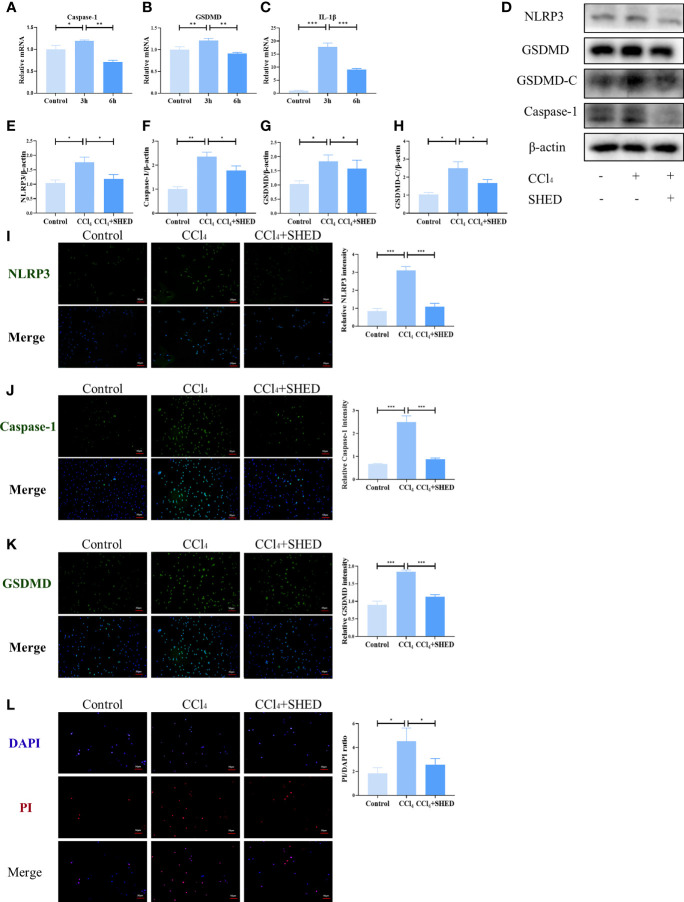
SHED inhibited CCl_4_-induced NLRP3 inflammasome activation and pyroptosis in hepatocytes. **(A–C)** The mRNA level of Caspase-1, GSDMD, and IL-1b was detected after 3h and 6h CCl_4_ stimulation analyzed by RT-qPCR. **(D–H)** The expression levels of NLRP3, Caspase-1, and GSDMD-C in hepatocytes in control, CCl_4_, and SHED infusion group by Western blotting analysis. **(I–K)** THe expression of NLRP3, Caspase-1 and GSDMD in hepatocytes in control, CCl_4_, and CCl_4_+SHED groups was analyzed by immunofluorescence staining. **(L)** The ratio of dead cells was determined by PI staining in control, CCl_4_, and CCl_4_+SHED groups. Scale bar: 50 μm. *p < 0.05, **p < 0.01, and ***p < 0.001. Graph bars show the mean ± SD. All of the assays were performed in triplicate. PI, propidium iodide; SHED, stem cells from human exfoliated deciduous teeth; GSDMD, gasdermin D.

The expression of GSDMD and Caspase-1 in mouse hepatic cell line NCTC 1469 was significantly upregulated after being stimulated with CCl_4_ for 3 h, as assessed by qPCR (**Figures 3A, B**). The expression of inflammatory cytokine IL-1β was also increased after CCl_4_ treatment (**Figure 3C**). The expression of NLRP3, Caspase-1, and GSDMD-C was significantly elevated after CCl_4_ stimulation, which was inhibited when co-cultured with SHED in the transwell system (**Figures 3D–H**). Immunofluorescence results showed that CCl_4_ activated the expression of NLRP3, Caspase-1, and GSDMD to increase in hepatocytes, while SHED treatment with a transwell system significantly downregulated their expression (**Figures 3I–K**). Compared with the control group, the CCl_4_ group showed more dead cells positive for PI staining, while SHED treatment decreased the number of PI-positive dead hepatocytes in the lower chamber, which indicated that SHED suppressed the pyroptosis of hepatocytes (**Figure 3L**).

### Stem Cells From Human Exfoliated Deciduous Teeth Inhibited CCl_4_-Induced Pyroptosis by Reducing Reactive Oxygen Species

**Figure 4:**
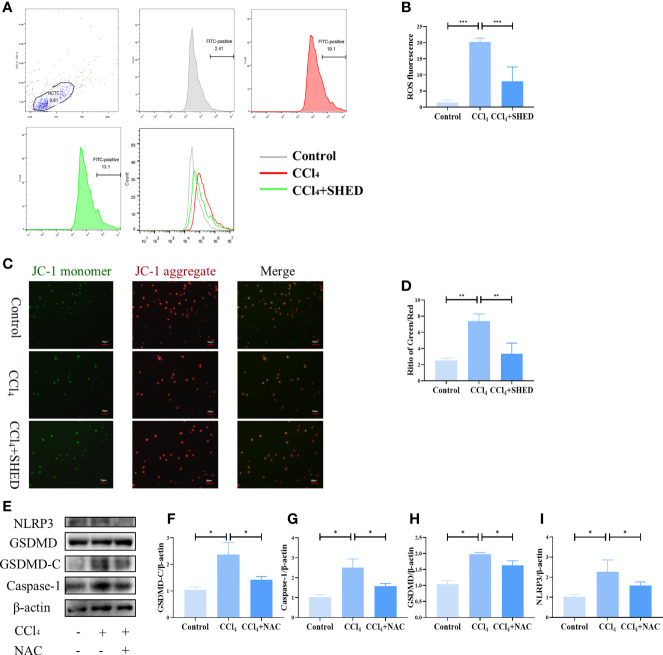
SHED attenuated CCl_4_-induced pyroptosis of hepatocytes by reducing ROS. **(A, B)** The intracellular ROS of hepatocytes in control, CCl_4_, and CCl_4_+SHED groups was analyzed by flow cytometric analysis. **(C, D)** MMP was measured by JC-1 staining of hepatocytes in control, CCl_4_, and CCl_4_+SHED groups. **(E–I)** The expression of NLRP3, Caspase-1, and GSDMD-C in hepatocytes in control, CCl_4_, and CCl_4_+NAC groups analyzed by Western blotting analysis. Scale bar: 50 μm. *p < 0.05, **p < 0.01, and ***p < 0.001. Graph bars show the mean ± SD. All of the assays were performed in triplicate. MMP, mitochondrial membrane potential; ROS, reactive oxygen species; NAC, antioxidant; JC-1, carbocyanine iodide; SHED, stem cells from human exfoliated deciduous teeth.

ROS is considered to play a role in pyroptosis (29). The production of ROS in hepatocytes was increased after CCl_4_ treatment, as assessed by flow cytometry analysis (**Figures 4A, B**). A decrease in MMP after CCl_4_ stimulation was detected by the ratio of green and red fluorescence, as assessed by the JC-1 MMP detection kit (**Figures 4C, D**). After being treated with SHED in transwell, ROS production was downregulated, and MMP was restored (**Figures 4A–D**). To analyze the effects of ROS on hepatocyte pyroptosis, antioxidant [*N*-acetylcysteine (NAC)], a ROS inhibitor, was used to treat hepatocytes. The results showed that the elevated expression of NLRP3, Caspase-1, and GSDMD-C induced by CCl_4_ treatment was suppressed by NAC treatment (**Figures 4E–I**). These data showed that SHED attenuated CCl_4_-induced pyroptosis of hepatocytes by reducing ROS.

### Pyroptosis Inhibitor Disulfiram Attenuated CCl_4_-Induced Liver Cirrhosis

**Figure 5:**
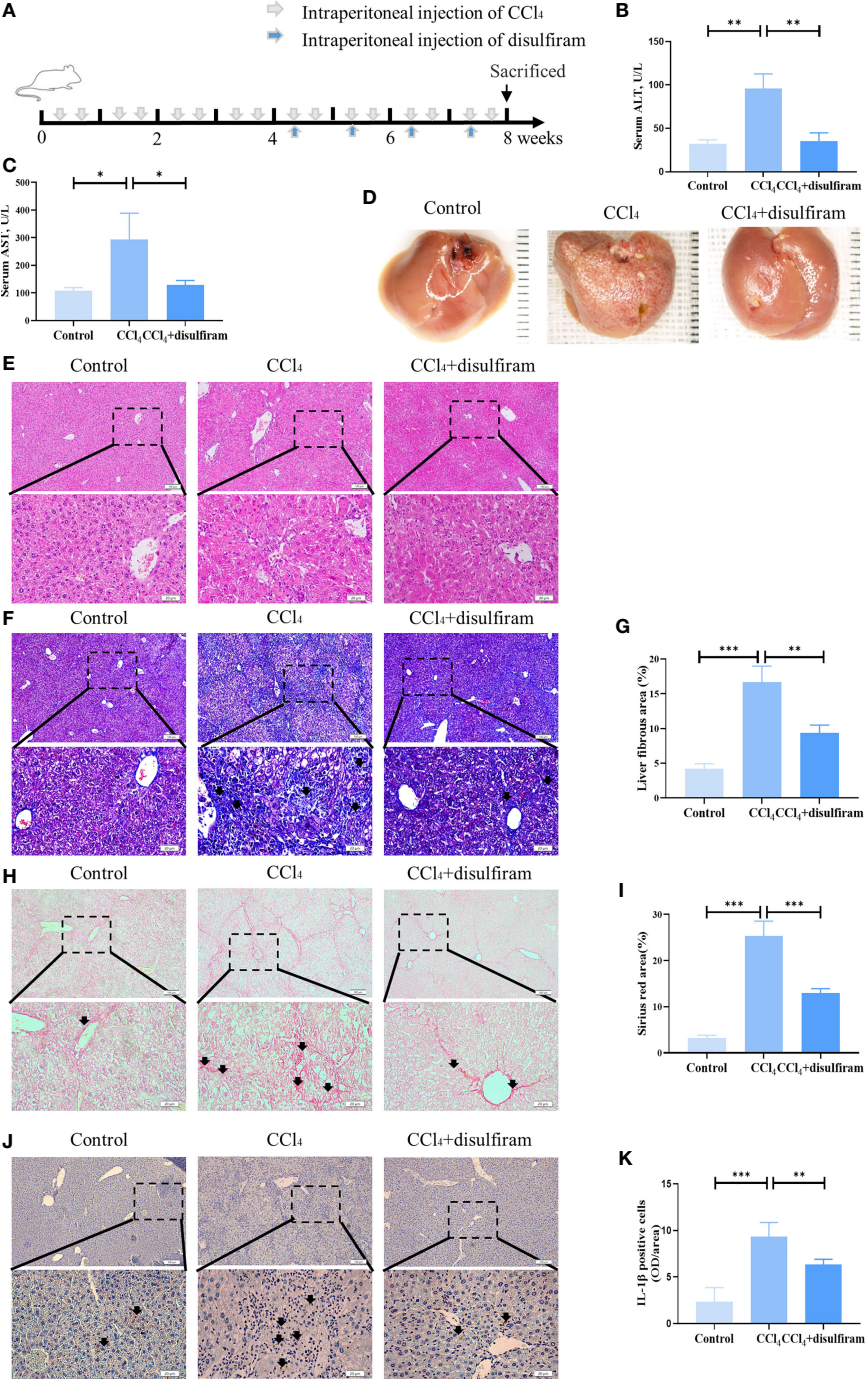
Pyroptosis inhibitor disulfiram attenuated CCl_4_-induced liver cirrhosis. **(A)** The schema of CCl_4_ and disulfiram injection to induce liver cirrhosis and treatment in mice. **(B, C)** The levels of serum ALT and AST from control, CCl_4_, and disulfiram-injected mice. **(D)** The gross appearance of the liver tissues from control, CCl_4_, and disulfiram-injected groups. **(E, F)** H&E staining of liver tissues from control, CCl_4_, and disulfiram-injected groups. **(F, G)** The collagen deposition in livers from control, CCl_4_, and disulfiram treatment groups, as assessed by Masson trichrome staining. Arrowheads, fibrous deposition. **(H, I)** Fibrous septum in livers from control, CCl_4_, and CCl_4_ mice with SHED infusion groups, as assessed by Sirius red staining. Arrowheads, fibrous septum. **(J, K)** The expression of IL-1β in control, CCl_4_, and disulfiram treatment groups analyzed by immunohistochemical staining. Scale bar: 100 and 20µm. *p < 0.05, **p < 0.01, and ***p < 0.001. Graph bars show the mean ± SD. All of the assays were performed in triplicate. ALT, alanine aminotransferase; AST, aspartate aminotransferase; SHED, stem cells from human exfoliated deciduous teeth.

**Figure 6:**
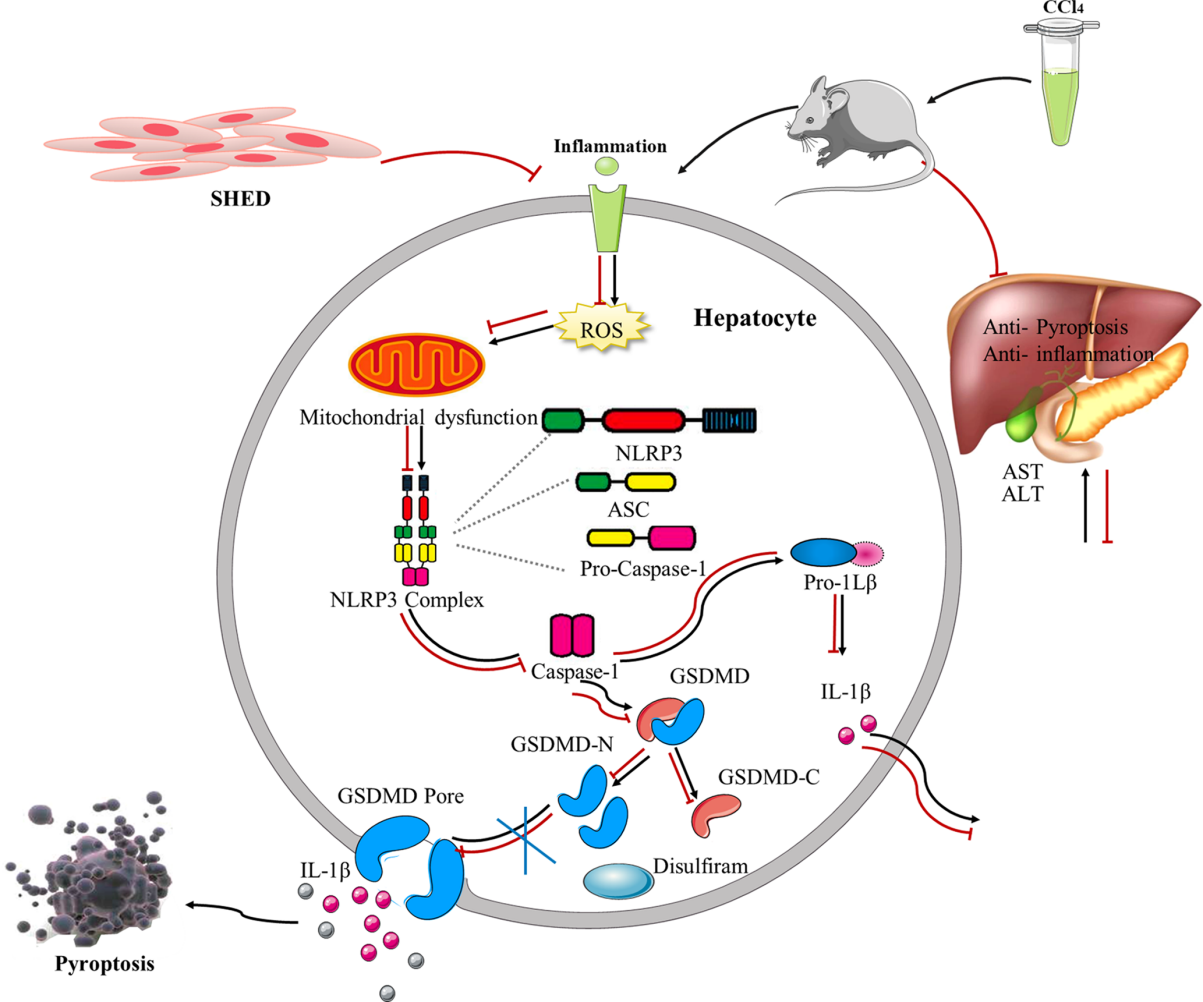
The schematic shows SHED alleviated CCl_4_-induced liver cirrhosis *via* inhibiting GSDMD-mediated pyroptosis in hepatocytes. SHED, stem cells from human exfoliated deciduous teeth; GSDMD, gasdermin D.

To verify the effects of pyroptosis on liver cirrhosis, we used disulfiram, a potent inhibitor of GSDMD (30), to treat liver cirrhosis (**Figure 5A**). The results showed that the levels of ALT and AST in mouse serum were decreased after disulfiram administration compared with the CCl_4_ group (**Figures 5B, C**). The liver fibrosis was decreased, and the gross morphology of the liver was improved after disulfiram treatment compared with the CCl_4_ group (**Figure 5D**). The disorganized liver cells arrangement, swelling cells, and inflammation in liver tissue were decreased in the disulfiram treatment group, as assessed by H&E staining (**Figure 5E**). The collagen deposition was also decreased in Masson trichrome staining in the disulfiram group when compared with the CCl_4_ group (**Figures 5F, G**). Compared with the CCl_4_ group, there were fewer fibrous septa formed in the disulfiram treatment group (**Figures 5H, I**). With the treatment of disulfiram, the expression of IL-1β was decreased after disulfiram treatment compared with the CCl_4_ group (**Figures 5J, K**). These results implied an important role of pyroptosis in the pathogenesis of liver cirrhosis and suggested a potential therapeutic strategy of targeting GSDMD-mediated pyroptosis in liver cirrhosis treatment (**Figure 6**).

## Discussion

In this study, we showed that SHED could attenuate liver cirrhosis. The important finding from this study is that GSDMD-induced pyroptosis was involved in murine liver cirrhosis. Moreover, SHED could suppress pyroptosis and protect liver cells from injury. The expression of GSDMD and Caspase-1 was increased in the liver cirrhosis group. The inhibitor of GSDMD, disulfiram, showed therapeutic effects for liver cirrhosis similar to SHED infusion. These data suggest that GSDMD plays an important role in the process of liver cirrhosis and may be a potential target for new therapeutic strategy exploration.

GSDMD is recently reported to execute programmed necrosis in inflammation, and GSDMD-formed pores allow inflammatory cytokine IL-1β release. IL-1β has been well studied to be an important pro-inflammatory cytokine that drives the pathogenesis of liver inflammation, fibrosis, and injury (31). In this study, we found that the IL-1β release was increased after CCl_4_ treatment. The maturation of pro-inflammatory cytokines such as pro-IL-1β and pro-IL-18 was mediated by the activation of Caspase-1. NLRP3 binds to pro-Caspase-1 and subsequently activates Caspase-1 (32). GSDMD is found to be a downstream molecule of Caspase-1 and cut into cleaved N-terminal GSDMD (GSDMD-N) and cleaved C-terminal GSDMD (GSDMD-C). GSDMD-N is identified to be the effective form to trigger pyroptosis by punching pores in the cell membrane, and it is inhibited by GSDMD-C when they are combined together (33). GSDMD-C also mediates the identification process of caspases and GSDMD, and the Caspase–GSDMD-C complex promotes dimerization-mediated caspase activation, rendering a cleavage independently of the cleavage-site tetrapeptide sequence (34). Matured IL-1β and IL-18 leak from the pores and amplify the inflammatory response. Here we showed that hepatocyte undergoing pyroptosis was mediated by NLRP3 in liver cirrhosis. It is reported that NLRC4 inflammasome was found to mediate the pyroptosis of hepatocytes in NASH, while NLRP3 knockout mice were not protected against hepatocyte pyroptosis (21). Caspase-11-mediated pyroptosis of hepatocytes was also reported to be involved in alcoholic hepatitis (35). Whether NLRC4 or Caspase-11 participates in liver cirrhosis needs further investigation.

GSDMD is a member of a family of conserved proteins that include gasdermin A, B, C, D, and E and DFNB59 (33), and most of them have now been shown to have pore-forming activity. GSDMA is expressed in epithelial cells and has been linked to autoimmune diseases and cancer (36).GSDMB is reported to be associated with asthma and colitis (37). GSDME is reported to promote inflammation and fibrosis in obstructive nephropathy (38). Recent studies demonstrate that the function of the gasdermin family still remains explored, and there are pathways of pyroptosis waiting to be discovered (39–41). The exact role of the GSDM family members and pathways of pyroptosis in liver cirrhosis still needs further exploration.

Disulfiram has been used as a treatment for alcoholism and proved relatively safe and is well tolerated by most patients (42). Recently, it was found to block pyroptosis and cytokine release in cells by inhibiting GSDMD pore formation, while it did not substantially inhibit inflammasome activation and GSDMD processing in cells (32). In our experiment, we found that disulfiram could improve liver dysfunction similar to SHED infusion, which verified the role of pyroptosis in liver fibrosis and implied the potential therapeutic targets.

Abnormal mitochondrial functions are linked to multiple diseases including liver fibrosis (43). Acute damage can trigger the permeabilization of mitochondrial membranes to initiate apoptosis or necrosis (44). Mitochondria house their own genome organized into DNA–protein complexes, the mitochondrial nucleoids. Exposed to the ROS, mitochondrial DNA (mtDNA) is at risk for oxidative damage and leaks into the cytosol, which may trigger inflammasome pyroptotic cell death (45) (46). We found that the cellular ROS was increased and the MMP was decreased in CCl_4_-induced hepatocytes, which indicated that CCl_4_ stimulation caused the dysfunction of mitochondria. NLRP3 inflammasome is reported to sense mitochondrial dysfunction (47), and it might cause the activation of the downstream pyroptosis pathway in hepatocytes. When treated with SHED, the ROS generation was suppressed and MMP returned to a relatively normal level. When treated with NAC, a ROS inhibitor, the pyroptosis pathway was also inhibited. We showed that SHED could decrease the ROS generation and restore the mitochondria function to protect hepatocytes from pyroptosis.

In this study, SHED infusion displayed an anti-pyroptosis effect in transwell co-culture experiments, indicating that this effect may be mediated by paracrine secretory factors. It has been reported that MSCs can secrete lots of molecules, such as soluble proteins, lipids, and extracellular vesicles (EVs). MSC-sourced secretome shows profound immunomodulatory and anti-inflammatory effects in a variety of diseases. Anti-inflammatory cytokines such as tumor necrosis factor-alpha (TNF-α) and IL-10 are reported to inhibit the proliferation of hepatic stellate cells and decrease collagen synthesis (48, 49). Hepatocyte growth factor-1 (HGF-1) and vascular endothelial growth factor (VEGF) secreted by MSCs can stabilize the barrier function of endothelial cells (50), which may reduce the damage to liver tissue. Recent studies have shown that exosomes are one of the key secretory products of MSCs mediating cell-to-cell communication. Exosome cargo consists of proteins, microRNA (miRNA), mRNA, and mitochondria (51). MSC-derived exosomes containing miR-542-3p are reported to reduce the expression of ROS and induce cell inflammatory response *via* inhibiting TLR4 (52), and the mitochondrial transfer was also reported to play a role in the therapeutic restoration of mitochondrial function by MSC-derived exosomes (53). The exact molecules that displayed the anti-pyroptosis effect in SHED need further investigation. Early studies indicated that SHED may promote liver tissue repair *via* differentiating into hepatocyte-like cells (14). By tracing SHED with DiR and CFSE labels, our study showed that SHED was recruited in the liver 1 day after transplantation and decreased in a time-dependent manner (16). It is reported that the apoptosis of the infusion MSCs increased in a time-dependent manner and display its immunosuppressive by recruiting T cells for FasL-mediated apoptosis or inducing indoleamine 2,3-dioxygenase (IDO) production in recipient phagocytes (54, 55). Autophagy of MSCs was also reported to be induced by a liver fibrosis environment (56). These results indicated that the therapeutic effects of SHED for liver cirrhosis may be dependent on the synergism of paracrine molecules and differentiating into hepatocyte-like cells. However, the precise mechanism for MSC infusion to treat liver cirrhosis needs further investigation.

## Conclusion

In summary, the present study demonstrated that SHED infusion attenuated liver damage by inhibiting the GSDMD-executed pyroptosis pathway in CCl_4_-induced liver cirrhosis. SHED treatment inhibited inflammasome NLRP3 activation by reducing ROS generation. Moreover, the pyroptosis inhibitor disulfiram also showed therapeutic effects for liver cirrhosis. SHED infusion and the targets on GSDMD may be potential novel therapeutic strategies for liver cirrhosis.

## Data Availability Statement

The original contributions presented in the study are included in the article/supplementary material. Further inquiries can be directed to the corresponding authors.

## Ethics Statement

The animal study was reviewed and approved by the Ethics Committee of Peking University.

## Author Contributions

PC and Y-kZ contributed to the collection and assembly of data, data analysis and interpretation, and manuscript drafting. C-sH, Y-mW, Z-mZ, SL, and L-jC contributed to the animal study. R-lY and J-hJ contributed to the overall design of the study, critical editing of the manuscript, and financial support. All authors read and approved the final manuscript.

## Funding

This work was supported by the National Natural Science Foundation of China No. 81970940 (R-lY), Ten-thousand Talents Program QNBJ-2020 (R-lY), and the National Science and Technology Major Project of the Ministry of Science and Technology of China No. 2018ZX10302207.

## Conflict of Interest

The authors declare that the research was conducted in the absence of any commercial or financial relationships that could be construed as a potential conflict of interest.

## Publisher’s Note

All claims expressed in this article are solely those of the authors and do not necessarily represent those of their affiliated organizations, or those of the publisher, the editors and the reviewers. Any product that may be evaluated in this article, or claim that may be made by its manufacturer, is not guaranteed or endorsed by the publisher.
